# Synthesis and Tribological Characterization of Ti_3_SiC_2_/ZnO Composites

**DOI:** 10.3390/ma14206088

**Published:** 2021-10-14

**Authors:** Rui Zhang, Wei Feng, Qi Wei, Shuai Ma

**Affiliations:** 1 School of Mechanical Engineering, Chengdu University, Chengdu 610106, China; fengwei@cdu.edu.cn; 2 School of Mechanical Engineering, Xinjiang University, Urumqi 830000, China; mashuai@stu.xju.edu.cn; 3 State Key Laboratory of Solid Lubrication, Lanzhou Institute of Chemical Physics, Chinese Academy of Sciences, Lanzhou 730000, China; qwei@licp.cas.cn

**Keywords:** Ti_3_SiC_2_/ZnO, spark plasma sintering, self-lubricating, tribo-oxidation, wear surface

## Abstract

Dense Ti_3_SiC_2_/ZnO composites were sintered at different temperatures by spark plasma sintering (SPS). The effects of sintering temperature on composition and mechanical properties of Ti_3_SiC_2_/ZnO composites were studied. The tribological behaviors of Ti_3_SiC_2_/ZnO composites/Inconel 718 alloy tribo-pairs at elevated temperature from 25 °C to 800 °C were discussed. The experimental results showed that the initial decomposition temperature of the Ti_3_SiC_2_/ZnO composite was 1150 °C, and Ti_3_SiC_2_ decomposed into TiC. When the decomposition temperature was higher than 1150 °C, the compositions of the Ti_3_SiC_2_/ZnO composites were Ti_3_SiC_2_, ZnO, and TiC. It was found that Ti_3_SiC_2_/ZnO composites had better self-lubricating performance than Ti_3_SiC_2_ at elevated temperature from 600 °C to 800 °C, which was owing to material transfers of tribo-pairs and sheared oxides generated by tribo-oxidation reactions.

## 1. Introduction

Ti_3_SiC_2_ is a ternary compound of the M_n_AX_n + 1_(MAX) phase which combines the merits of both metals and ceramics, such as low density, high elastic modulus, favorable electrical conductivity, excellent high-temperature oxidation resistance, and good mechanical properties [1,2,3,4]. More significantly, it has potential application in self-lubricating under certain conditions [5,6].

With the development and innovation of industrial machinery, lubrication under extreme conditions has become one of the most interesting research areas [7,8,9,10]. Some traditional solid lubricants, such as graphite and MoS_2_, are not able to work effectively at elevated temperature in the fields of aerospace, aircraft, and others. Therefore, scholars have tried to prepare advanced materials for continuous lubrication in a wide-temperature range [11,12,13].

In previous studies, Ti_3_SiC_2_/Pb composites were successfully prepared, and it was found that the addition of Pb could not only improve mechanical properties, but also significantly decreased the friction coefficient and wear rate at 600–800 °C [14,15]. H.J. Wang et al. used SPS to synthesize Ti_3_SiC_2_/Al_2_O_3_ composites and they found that the mechanical properties and friction-wear properties of Ti_3_SiC_2_ could also be improved by adding Al_2_O_3_ [12,16,17]. Islak et al. [18] prepared Ti_3_SiC_2_ and Ti_3_SiC_2_/SiC composites by a mixture of Ti, Si, TiC, and Al powder and studied their mechanical properties and tribological performances, respectively. It was shown that Ti_3_SiC_2_/SiC composites had higher hardness, fracture toughness, and wear resistance than Ti_3_SiC_2_ [19,20]. These studies showed that the mechanical properties and tribological performance of Ti_3_SiC_2_ were relatively poor at elevated temperature. Fortunately, the friction coefficient and wear rate could be reduced with the incorporation of second phases.

Ternary layered carbide, Ti_3_SiC_2_, has good comprehensive properties. In this study, solid lubricant ZnO was used as the second phase to prepare Ti_3_SiC_2_/ZnO composites. It was expected that the addition of ZnO would improve the tribological performance of Ti_3_SiC_2_ and especially promote its lubrication at elevated temperatures (600–800 °C).

## 2. Materials and Methods

### 2.1. Samples Preparation

The composites were prepared from powder mixture of Ti_3_SiC_2_ (purity: >98%, average particle size: 2 μm, Jinhezhi Materials Ltd., Beijing, China) and ZnO (purity: >99.5%, average particle size: 1 μm, Xilong Chemical Ltd., Shantou, China). The volume fraction of ZnO in the Ti_3_SiC_2_/ZnO composite was 15 vol.%. The mixture with designed composition was firstly mixed for 6 h by ball-milling then filled into graphite dies (inner diameter: Φ25 mm) and sintered by SPS (manufacturer: Shanghai Chenhua Electric Furnace Co., Ltd., Shanghai, China, instrument model: SPS-20T10) under vacuum. The heating rate and pressure were 50 °C/min and 35 MPa, respectively. The holding time was 5 min at sintered temperatures (1100 °C, 1150 °C, and 1200 °C). Then, the furnace temperature was reduced to 1000 °C with a rate of 50 °C/min, and held at 1000 °C for 15 min. Subsequently, the sample was cooled to room temperature with the furnace. For comparison, Ti_3_SiC_2_ was prepared at 1200 °C (purity: >99.5%, average particle size: 1 μm).

### 2.2. Mechanical Property

Densities of the composites were determined by using Archimedes′ principle. Micro-hardness was measured on a Micro-hardness Tester (Hengyi Technology Co., Ltd., Shanghai, China, instrument model: MH-5-VM) applying a load of 500 g with a dwell time of 10 s. Flexural strength and compression strength were measured using a universal material tester (SANS-CMT5205, MTS China, Shenzhen, China). The three-point bending test was conducted to obtain the flexural strength and the samples were cut to 3 mm high × 4 mm wide × 20 mm long. The cross-head speed and span were 0.05 mm/min and 16 mm, respectively. The sizes of the samples for the compression strength test were *Φ* 5 mm × 12.5 mm and the cross-head speed was 0.2 mm/min. All samples of mechanical properties were prepared by wire cutting.

### 2.3. Friction and Wear Test

The friction and wear tests were conducted on a high temperature tribometer with a pin-on-disk configuration (CSM Instruments SA, Peseux, Switzerland). Friction tests under each condition were repeated three times. The pin was a Ti_3_SiC_2_ and Ti_3_SiC_2_/ZnO composite with a size of *Φ* 6 mm × 12 mm by wire cutting. The disk was Inconel 78 alloy with a size of *Φ* 32 mm × 8 mm by wire cutting. The surface roughness (Ra) of the disk was 0.06 μm after grinding and polishing. The test was conducted at room temperature with relative humidity in the range of 20–50%. The sliding speed was 0.5 m/s and the normal load was 5 N. The sliding distance was 200 m. The friction coefficient was be recorded by a computer during the test. The wear volume was calculated by measuring the volume loss of the Ti_3_SiC_2_ and Ti_3_SiC_2_/ZnO pin and the cross-sectional area of the wear track on Inconel 718 disk using optical microscopy and 3D surface profilometry. The wear rates were calculated from the wear volume divided by sliding distance and load. In addition, scanning electron microscope (SEM, JEOL Company, Tokyo, Japan) was used to observe the wear surface morphology, X-ray diffraction (XRD, Rigaku Corporation, Tokyo, Japan) was used to analyze the phase of the wear surface, and X-ray photoelectron spectroscopy (XPS, Physical Electronics Corporation, Chanhassen, MN, USA) was used to analyze the oxidation on the wear surface.

## 3. Results and Discussion

### 3.1. Effects of Sintering Temperature on Composition in the Ti_3_SiC_2_–ZnO System

**Figure 1:**
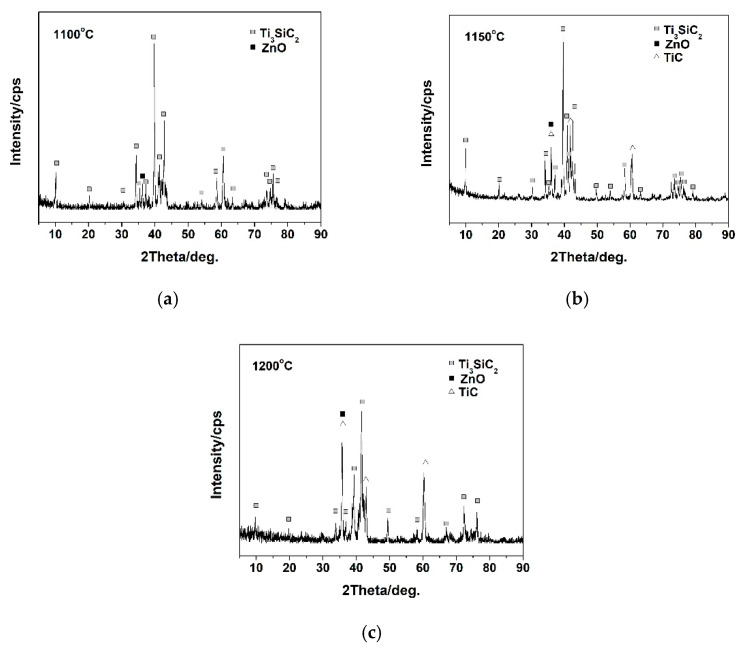
XRD pattern for prepared Ti_3_SiC_2_/ZnO composites at different sintering temperatures: (**a**) 1100 °C, (**b**) 1150 °C, and (**c**) 1200 °C.

**Figure 2:**
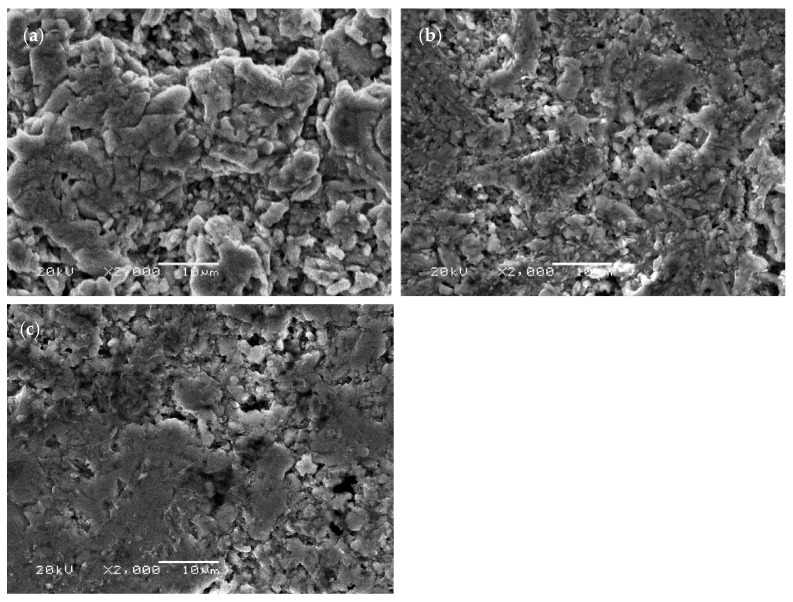
Micrographs of Ti_3_SiC_2_/ZnO composites at different sintering temperature: (**a**) 1100 °C, (**b**) 1150 °C, and (**c**) 1200 °C.

Figure 1 shows the XRD patterns of Ti_3_SiC_2_/ZnO composites at different sintering temperatures. The XRD pattern at 1100 °C shows that the main components of sintered samples were Ti_3_SiC_2_ and ZnO. The results indicate that no chemical reaction occurred at 1100 °C between Ti_3_SiC_2_ and ZnO, and Ti_3_SiC_2_ did not decompose. At 1150 °C and 1200 °C, diffraction peaks of Ti_3_SiC_2_, TiC and ZnO were found in the XRD pattern, which indicated that TiC was generated by partial decomposition of Ti_3_SiC_2_. This result was consistent with the previous research results [15]. Figure 2 shows the micrographs of Ti_3_SiC_2_/ZnO composites at 1100 °C, 1150 °C, and 1200 °C. As seen from the figure, at 1100 °C, the grain of Ti_3_SiC_2_/ZnO composites is coarse and irregular in shape, and many pores are distributed among them. With the increase of temperature, the grain size was refined, and its pores were obviously reduced.

### 3.2. Effects of Sintering Temperature on the Mechanical Properties of the Ti_3_SiC_2_/ZnO Composites

**Figure 3:**
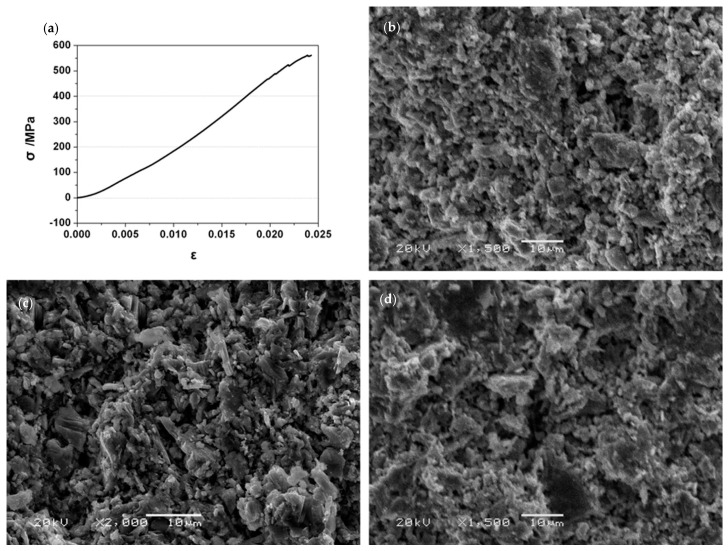
(**a**) Compressive stress-strain curve of the Ti_3_SiC_2_–ZnO composite, and the cross-section map after the three-point bending test with different sintering temperature: (**b**) 1100 °C, (**c**) 1150 °C, and (**d**) 1200 °C.

Table 1 shows some mechanical properties of the Ti_3_SiC_2_ and Ti_3_SiC_2_/ZnO composites at different sintering temperatures. When the sintering temperature was 1100 °C, the temperature was so low that it caused the formation of coarse grains and many holes in the composites with poor mechanical properties. Therefore, these results were not listed in the table. Compared with Ti_3_SiC_2_, the relative density, microhardness, flexural strength, and compression strength of the Ti_3_SiC_2_/ZnO composites were decreased. In addition, with the increase of sintering temperature, the mechanical properties of the Ti_3_SiC_2_/ZnO composites had certain changes: the flexural strength decreased by about 15.4%, compression strength increased by about 23.5%, and Vickers hardness increased by only 3.7%. It can be concluded that Ti_3_SiC_2_/ZnO composites have relatively good mechanical properties with sintering at 1200 °C, and their microhardness, bending strength, and compression strength were 4.67 ± 0.93 GPa, 115 ± 5 MPa, and 557 ± 3 MPa, respectively. Figure 3 shows the compression stress-strain curve of the Ti_3_SiC_2_/ZnO composites sintered at a temperature of 1200 °C and the bending fracture morphology of the composites at different sintering temperatures. As seen from Figure 3a, the stress-strain curve of the Ti_3_SiC_2_/ZnO composites was close to a straight line with a certain slope before the fracture. Consequently, the composites only displayed elastic deformation before the fracture, which was consistent with the characteristic of brittle fracture. In addition, the fracture mechanism of Ti_3_SiC_2_ was also brittle fracture [15]. Moreover, the addition of ZnO had no obvious effect on the fracture mode. Figure 3b–d shows the bending fracture morphology of materials at different sintering temperatures. From the perspective of macroscopic morphology, the fracture mode of the Ti_3_SiC_2_/ZnO composites at room temperature was brittle fracture.

### 3.3. Tribological Behavior of Ti_3_SiC_2_/ZnO Composites

#### 3.3.1. Friction Coefficients and Wear Rates

As mentioned before, the Ti_3_SiC_2_/ZnO composites sintered at 1200 °C (named TSC-ZN) had favorable mechanical performance. The tribological and wear properties of TSC-ZN and Inconel 718 alloys were studied at 25–800 °C. In comparison, the tribological and wear properties of Ti_3_SiC_2_ sintered at 1200 °C (named TSC) were also tested.

**Figure 4:**
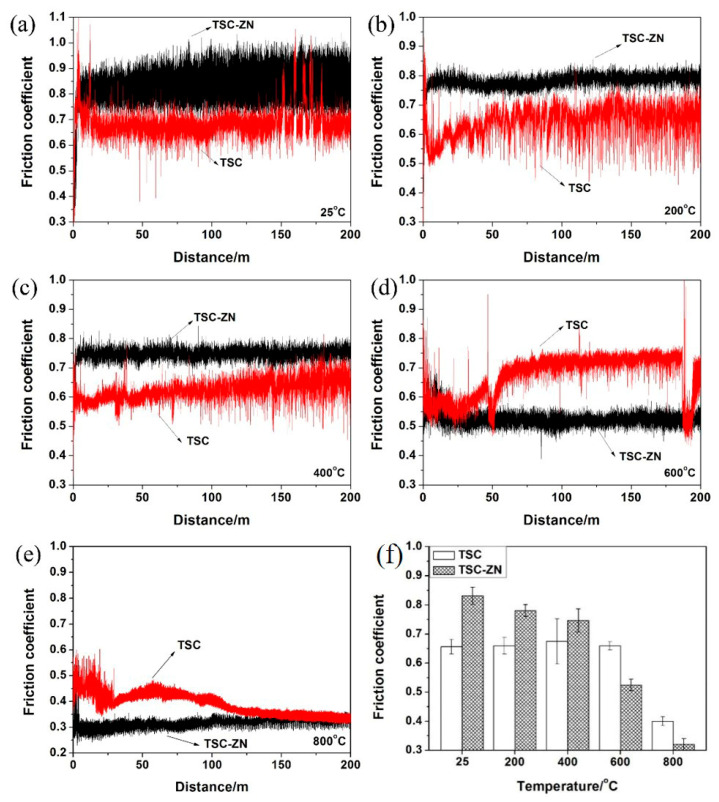
The friction coefficients and (**f**) average friction coefficients as a function of sliding distance and the average friction coefficients of Ti_3_SiC_2_ and Ti_3_SiC_2_/ZnO composites at (**a**) 25 °C, (**b**) 200 °C, (**c**) 400 °C, (**d**) 600 °C, and (**e**) 800 °C.

Figure 4 shows the friction coefficients of the Ti_3_SiC_2_ and Ti_3_SiC_2_/ZnO composites as a function of sliding distances and their average friction coefficients at different temperatures. From 25 °C to 400 °C, the friction coefficients of the Ti_3_SiC_2_/ZnO composites were higher than that of Ti_3_SiC_2_, and their friction coefficients fluctuated largely at 25 °C. However, the friction coefficients of the Ti_3_SiC_2_/ZnO composites were obviously lower than that of Ti_3_SiC_2_ from 600 °C to 800 °C. As seen from the figure, the friction coefficients of the Ti_3_SiC_2_/ZnO composites decreased gradually with the increase of temperature at 25–800 °C and there was a marked decreased at 600 °C. For Ti_3_SiC_2_, the friction coefficients were relatively stable at 25–600 °C, and they showed a significant decrease at 800 °C. In addition, the average friction coefficients of the Ti_3_SiC_2_/ZnO composites were higher than those of Ti_3_SiC_2_ at 25–400 °C, but they were lower at 600–800 °C. It could be concluded that Ti_3_SiC_2_ showed excellent self-lubricating properties at 800 °C, while the Ti_3_SiC_2_/ZnO composites showed excellent self-lubricating properties at 600 °C.

**Figure 5:**
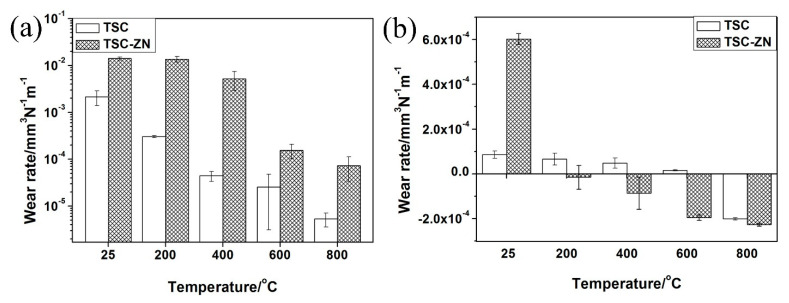
The wear rates for (**a**) pin and (**b**) disk of Ti_3_SiC_2_/Inconel 718 and Ti_3_SiC_2_–ZnO /Inconel 718 alloy tribo-pairs as a function of temperature.

Figure 5a shows the variation in wear rates as a function of the pin of the Ti_3_SiC_2_/Inconel 718 alloy and Ti_3_SiC_2_/ZnO-Inconel 718 alloy tribo-pairs at different temperatures. For the Ti_3_SiC_2_/ZnO-Inconel 718 alloy tribo-pair, no obvious change was found in the wear rates of the pin (Ti_3_SiC_2_/ZnO) at 25–400 °C and its wear rates decreased significantly at above 400 °C and reduced to about 7.32 × 10^−5^ mm^3^/N m at 800 °C. For the Ti_3_SiC_2_/Inconel 718 alloy tribo-pairs, the wear rates of the pin (Ti_3_SiC_2_) decreased gradually with the increase of temperature (25–800 °C) and reduced to about 5.34 × 10^−6^ mm^3^/N m at 800 °C. Figure 5b shows the variation in wear rates as a function of the disks of the Ti_3_SiC_2_/Inconel 718 alloy and Ti_3_SiC_2_/ZnO-Inconel 718 tribo-pairs at different temperatures. For the Ti_3_SiC_2_/ZnO-Inconel 718 alloy tribo-pair, the disk (Inconel 718 alloy) showed occured at 25 °C; however, it turned into negative wear above 200 °C. Additionally, with the increase of temperature, the negative wear rates increased gradually and reached about −2.27 × 10^−4^ mm^3^/N m at 800 °C. In addition, the wear rates of the disk (Inconel 718) of the Ti_3_SiC_2_/Inconel 718 alloy tribo-pair were positive at 25–600 °C and decreased with the increase of temperature. Then, its wear rates became negative at 800 °C and the negative wear rate were close to that of the disk (Inconel 718) of the Ti_3_SiC_2_/ZnO-Inconel 718 alloy tribo-pair.

The experimental results showed that the wear resistance of the Ti_3_SiC_2_/ZnO composites was worse than that of Ti_3_SiC_2_ from 600 °C to 800 °C; however, the friction coefficients of the Ti_3_SiC_2_/ZnO composites were lower than that of Ti_3_SiC_2_. Therefore, the Ti_3_SiC_2_/ZnO composites show good self-lubricating performance. It is feasible to add ZnO to improve the tribological performance of Ti_3_SiC_2_ at elevated temperatures.

#### 3.3.2. Morphology and Composition of the Worn Surfaces

**Figure 6:**
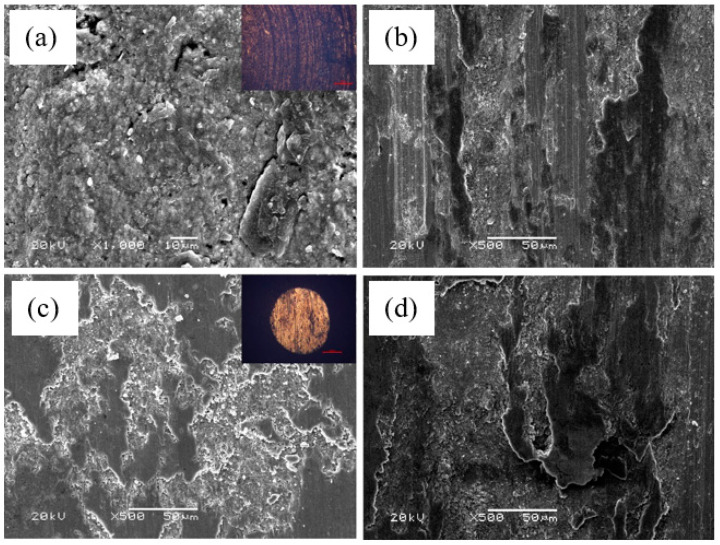
Typical SEM secondary electron images of the worn surface of the Ti_3_SiC_2_–ZnO/Inconel 718 alloy tribo-pair: (**a**) Ti_3_SiC_2_–ZnO at 25 °C, (**b**) Inconel 718 alloy at 25 °C, (**c**) Ti_3_SiC_2_–ZnO at 600 °C, and (**d**) Inconel 718 alloy at 600 °C.

Figure 6 showed the worn surfaces of the Ti_3_SiC_2_/ZnO-Inconel 718 alloy tribo-pair from 25 °C to 800 °C, which was the typical morphology of the worn pin and disk below 800 °C. It was seen that the wear mechanism caused by the pulling out and falling off of Ti_3_SiC_2_ grains during sliding was abrasive wear. There were some pits on the worn surfaces of the Ti_3_SiC_2_/ZnO composites, and the worn surfaces were relatively rough (see Figure 6a). At 25–400 °C, there was plastic flow on the worn surfaces of the Inconel 718 alloy (see Figure 6b), and Ti, Si, and Zn elements could be detected on these worn surfaces (see Table 2(b)), which indicated that there was a one-way transfer of materials from the Ti_3_SiC_2_/ZnO composites to the Inconel 718 alloy during the friction process. At 600–800 °C, Ti_3_SiC_2_ grains still fell off, and the Ti_3_SiC_2_/ZnO composites also exhibited certain characteristics of plastic flow (see Figure 6c). Similarly, the surfaces of the Inconel 718 alloy still had characteristics of plastic flow (see Figure 6d). Moreover, there was material transfer between tribo-pairs, and the material transfer films became denser after sintering at high temperatures.

In conclusion, there was a one-way transfer of materials from the Ti_3_SiC_2_/ZnO composites to the Inconel 718 alloy in the friction process at 25–400 °C, and a mutual transfer of materials between the Ti_3_SiC_2_/ZnO composites and the Inconel 718 alloy in the friction process at 600–800 °C.

#### 3.3.3. Competition of the Tribo-Oxides

**Figure 7:**
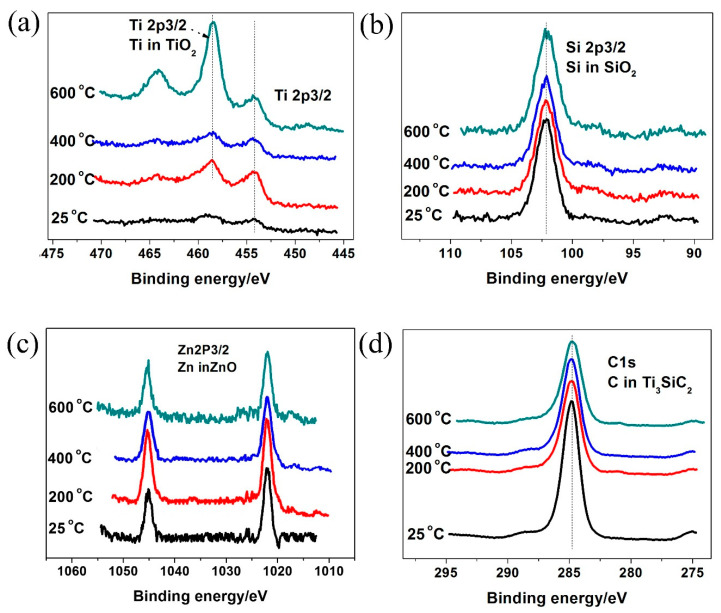
X-ray photoelectron spectroscopy for (**a**) Ti2p, (**b**) Si2p, (**c**) Zn2p, and (**d**) C1s on the worn surface of Ti_3_SiC_2_/ZnO composites after sliding against Inconel 718 alloy at different temperatures.

Figure 7 shows the element chemical states after friction and wear test from 25 °C to 600 °C. From the experimental results, it can be seen that ZnO and SiO_2_ were formed on the worn surface of the Ti_3_SiC_2_/ZnO composites at 25 °C. At 200–600 °C, the oxide films consisted of ZnO, SiO_2,_ and TiO_2_. As shown above, tribo-oxidation reactions occurred for the Ti_3_SiC_2_/ZnO composites to generate SiO_2_ and TiO_2_.

When the generation rate of oxide was greater than its consumption rate, an oxide layer was formed on the friction surface. The progress of the tribo-oxidation reaction depended on the frictional heat. At 25–400 °C, the oxidation reaction induced by friction heat was not enough to form an oxide film on the friction surface. Therefore, the tribological and wear properties of the Ti_3_SiC_2_/ZnO composites was not improved from 25 °C to 400 °C. However, when the temperature exceeded 600 °C, the friction heat provided enough energy to generate oxides continuously. At this point, the formed oxides, such as ZnO, SiO_2_, and TiO_2_, had obvious characteristics of plastic flow and could be sheared easily. In addition, the oxide films avoided direct contact between the Ti_3_SiC_2_/ZnO composites and the Inconel 718 alloy, and also decreased the material transfer and dropping of grains of Ti_3_SiC_2_. Therefore, the friction coefficients of the Ti_3_SiC_2_/ZnO composites decreased significantly. With the increase of temperature, the oxide films would be softened, making it easier to shear.

## 4. Conclusions

Dense Ti_3_SiC_2_/ZnO composites were successfully prepared by SPS from the mixture of Ti_3_SiC_2_ and ZnO powders. The friction coefficients and wear rates of the composites were relatively high at low and medium temperatures (at 25–400 °C). Fortunately, the friction coefficients of the composites decreased significantly at higher temperature (at 600–800 °C), due to material transfers of tribo-pairs and the presence of SiO_2_ and TiO_2_. These oxide films had obvious characteristics of plastic flow and avoided direct contact of tribo-pairs. Therefore, Ti_3_SiC_2_/ZnO composites could be used as a solid lubricant in a wide-temperature range.

## Figures and Tables

**Table 1:** Properties of Ti_3_SiC_2_ and Ti_3_SiC_2_/ZnO.

Sample	Relative Density (%)	Microhardness (GPa)	Flexural Strength (MPa)	Compression Strength (MPa)
Ti_3_SiC_2_	98.24	5.5 ± 0.2	428 ± 10	1230 ± 13
Ti_3_SiC_2_-15ZnO-1100	*	*	*	*
Ti_3_SiC_2_-15ZnO-1150	96.35	4.50 ± 0.87	136 ± 11	451 ± 12
Ti_3_SiC_2_-15ZnO-1200	97.32	4.67 ± 0.93	115 ± 5	557 ± 3

**Table 2:** Average phase chemical composition of Figure 6 as determined by EDS.

Position	Sample	Temperature (°C)	Atomic Percentage (at.%)
a	TSC-ZN pin	25	24.7%Ti, 6.3%Si, 45.4%C, 2.1%Al, 2.2%Zn, 19.3%O
c	TSC-ZN pin	200	28.5%Ti, 9.1%Si, 39.5%C, 3.0%Al, 2.2%Zn, 17.7%O
b	Inconel 718 disk	25	13.9%Ti, 5.8%Si, 37.9%C, 2.0%Al, 0.9%Zn, 24.5%O, 0.3%S, 7.5%Ni, 3.6%Cr, 3.2%Fe, 0.4%Nb
d	Inconel 718 disk	200	20.9%Ti, 8.3%Si, 36.6%C, 2.5%Al, 0.3%Zn, 25.3%O, 3.3%Ni, 1.4%Cr, 1.4%Fe

## Data Availability

The data presented in this study are available within the article.
